# Polystyrene nanoplastics mediate oxidative stress, senescence, and apoptosis in a human alveolar epithelial cell line

**DOI:** 10.3389/fpubh.2024.1385387

**Published:** 2024-05-10

**Authors:** Cristina Milillo, Eleonora Aruffo, Piero Di Carlo, Antonia Patruno, Marco Gatta, Annalisa Bruno, Melania Dovizio, Lisa Marinelli, Marilisa Pia Dimmito, Viviana Di Giacomo, Cecilia Paolini, Mirko Pesce, Patrizia Ballerini

**Affiliations:** ^1^Center for Advanced Studies and Technology (CAST), “G. d’Annunzio” University of Chieti-Pescara, Chieti, Italy; ^2^Department of Psychological, Health and Territorial Sciences, “G. d’Annunzio” University of Chieti-Pescara, Chieti, Italy; ^3^UdA-TechLab, Research Center, “G. d’Annunzio” University of Chieti-Pescara, Chieti, Italy; ^4^Department of Medicine and Aging Sciences, “G. d’Annunzio” University of Chieti-Pescara, Chieti, Italy; ^5^Department of Innovative Technologies in Medicine & Dentistry, “G. d’Annunzio” University of Chieti-Pescara, Chieti, Italy; ^6^Department of Pharmacy, “G. d’Annunzio” University of Chieti-Pescara, Chieti, Italy; ^7^Department of Neuroscience, Imaging and Clinical Sciences, “G. d’Annunzio” University of Chieti-Pescara, Chieti, Italy

**Keywords:** microplastics, nanoplastics, polystyrene, toxicity, oxidative stress, apoptosis, senescence, alveolar epithelial cells

## Abstract

**Background:**

Nanoplastics, an emerging form of pollution, are easily consumed by organisms and pose a significant threat to biological functions due to their size, expansive surface area, and potent ability to penetrate biological systems. Recent findings indicate an increasing presence of airborne nanoplastics in atmospheric samples, such as polystyrene (PS), raising concerns about potential risks to the human respiratory system.

**Methods:**

This study investigates the impact of 800 nm diameter-PS nanoparticles (PS-NPs) on A549, a human lung adenocarcinoma cell line, examining cell viability, redox balance, senescence, apoptosis, and internalization. We also analyzed the expression of hallmark genes of these processes.

**Results:**

We demonstrated that PS-NPs of 800 nm in diameter significantly affected cell viability, inducing oxidative stress, cellular senescence, and apoptosis. PS-NPs also penetrated the cytoplasm of A549 cells. These nanoparticles triggered the transcription of genes comprised in the antioxidant network [SOD1 (protein name: superoxide dismutase 1, soluble), SOD2 (protein name: superoxide dismutase 2, mitochondrial), CAT (protein name: catalase), Gpx1 (protein name: glutathione peroxidase 1), and HMOX1 (protein name: heme oxygenase 1)], senescence-associated secretory phenotype [Cdkn1a (protein name: cyclin-dependent kinase inhibitor 1A), IL1A (protein name: interleukin 1 alpha), IL1B (protein name: interleukin 1 beta), IL6 (protein name: interleukin 6), and CXCL8 (protein name: C-X-C motif chemokine ligand 8)], and others involved in the apoptosis modulation [BAX (protein name: Bcl2 associated X, apoptosis regulator), CASP3 (protein name: caspase 3), and BCL2 (protein name: Bcl2, apoptosis regulator)].

**Conclusion:**

Collectively, this investigation underscores the importance of concentration (dose-dependent effect) and exposure duration as pivotal factors in assessing the toxic effects of PS-NPs on alveolar epithelial cells. Greater attention needs to be directed toward comprehending the risks of cancer development associated with air pollution and the ensuing environmental toxicological impacts on humans and other terrestrial mammals.

## 1 Introduction

Plastic is one of the most convenient existing materials and is widely used worldwide. Counting statistics, the global production of plastics in Europe in 2021 has passed 58 million tons; thus, plastic waste is considered one of the main environmental challenges (1). Plastics are released into the environment constantly and differently and in large amounts. They exist in different environmental media for a long time and gradually degrade into microplastics (MPs) with a diameter smaller than 5 mm through different mechanisms, including fragmentation, biological degradation processes, and chemical aging (2). During and after these processes, they are widely distributed in the atmosphere (3).

The world distribution of MPs in the atmosphere seems to be quite ubiquitous. Many studies reported that quantifiable concentrations of MPs are detectable in the atmosphere of remote and uninhabited Artic regions (4), desolated mountain regions, such as French Pyrenees (5), and Rocky Mountain National Park in USA (6), whereas elevated concentrations have been measured in the atmosphere of the greatest cities of the world, such as in Asia (7).

In the atmosphere, the decomposition process continues through the reduction of the particles’ size in a time-dependent manner, leading to the formation of nanoplastics (NPs), defined as particles 1 nm ≤ x ≤ 1 μm (2). Their rapid accumulation in the air is mainly responsible for the resulting global pollution because they are more concentrated than MPs, and due to the lower dimension, they are easier to transport at long distances in the atmosphere (8).

The contact of airborne plastics with the respiratory tract by inhalation represents one of the prime routes of human exposition to air pollution. In this scenario, the lung is the most relevant potential target of air pollutants and airborne plastics. Due to their size and structure, MPs/NPs can enter the lungs (9), and recently, it has been shown that continuous exposition to MPs/NPs can induce potential respiratory occupational diseases, thus contributing to the induction of lung cancer (3, 7, 10). However, compared with soil and water, the quantitative analysis of MPs/NPs in the atmosphere and the effects of their inhalation on human health are still poorly investigated.

Currently, there are at least 45 different commercially available plastics, including polypropylene, polyethylene, polyethylene terephthalate, and polystyrene (PS). The latter represents more than 5% of the global demand for plastic and has been widely used in food containers, stuff packages, building insulation, inner liners for refrigerators, eyeglass frames, personal care products, and electronic goods (1, 11, 12).

The high diffusion of PS is due to its attractive features, such as low cost, low density, clarity, dimensional stability, and adaptability to radiation sterilization. PS is used in many medical and biomedical applications. In particular, it is present in thermoformed products such as catheters, heart pumps, and epidural trays, as well as in labware such as Petri dishes and other disposables for tissue culture. In addition, PS is also used in respiratory care equipment, syringe hubs, and suction canisters, and it is competitive with polypropylene and acrylics in labware and packaging for kits and trays (13).

Interestingly, PS is detected in the particulate matter (PM), a suspended combination of solid particles and liquid droplets, which, based on size, is divided into two main fractions containing particles with an aerodynamic diameter equal to or less than 2.5 (PM2.5) or 10 μm (PM10). These fractions have been recently reported to be 2.09 and 1.81 ng/m^3^ (14). Since PM2.5 can penetrate the respiratory tract and reach the circulatory system, it can cause human health problems, including different types of cancers (15, 16).

As reported above, NPs have smaller dimensions, larger specific surface area, and more substantial biological permeability than MPs. Consequently, they are easier to enter cells through biofilm, destroy organelles, interfere with cell homeostasis, and cause tissue injury (17). Interestingly, NPs can absorb different kinds of harmful environmental compounds, such as polycyclic aromatic hydrocarbons and heavy metals, thus exerting even more severe toxicity once inside the body (18).

Particles sized less than 10 μm enter the lungs and can reach the alveolar cells, which accumulate these air pollutants over a lifetime (19). A recent study found that PS-NPs could derange the lung cell barrier and affect normal lung function (20), whereas Xu et al. (21) showed that PS-NPs of 25 and 70 nm in diameter significantly modify the cell cycle in human lung adenocarcinoma cell line A549 cells, considered a representative *in vitro* model of the human alveolar epithelium (22). In the same cellular model, Shi et al. (23) found decreased metabolic cellular activity and increased inflammation when the cells were simultaneously exposed to PS-NPs (100 nm) and the organic pollutant phthalate. Goodman et al. (24) studied the effects of PS-MPs 1 or 10 μm, resembling particle size found in the atmosphere (25, 26) on A549 cells, showing that they caused inhibition of cell proliferation and major changes in cell morphology. In the present study, we investigated the potential toxicity of PS-NPs (800 nm) on A549 cells, which is close to this size range, but it remains largely unexplored (27). To this aim, we assessed the effects of PS-NP exposure on cell viability, the production of reactive oxygen species (ROS), antioxidant network activity, apoptosis occurrence, senescence onset, and PS-NP internalization uptake. The results of this study contribute to clarifying the mechanisms potentially involved in the toxicity elicitation by primary exposition to PS-NPs on the respiratory tract.

## 2 Materials and methods

### 2.1 Materials

The human lung adenocarcinoma cell line A549 and all the components used for cell cultures were purchased from Sigma-Aldrich (Milan, Italy). The CellTiter 96® AQueous One Solution Cell Proliferation Assay and ROS-Glo^TM^ H_2_O_2_ Assay were purchased by Promega (Madison, MI, United States). All components used for RNA extraction and RT-PCR, including TaqMan primers, were purchased from Invitrogen, Life Technologies (California, United States). The Bradford Assay reagent and iScript cDNA synthesis kit were purchased from Bio-Rad (Hercules, CA, United States). Nanoparticles based on polystyrene (800 nm) (PS-NPs) were obtained from Sigma-Aldrich (Milan, Italy).

### 2.2 Cell culture

A549 cells were grown in Dulbecco’s Modified Eagle Medium-High Glucose with phenol red (DMEM, Sigma-Aldrich) supplemented with 10% (v/v) heat-inactivated fetal bovine serum, 100 U/mL penicillin, 100 μg/mL streptomycin, and 1 mM sodium pyruvate. Cell culture was carried out in a humidified atmosphere of 5% CO_2_ at 37°C. When confluent, cells were detached enzymatically with trypsin-ethylenediamine tetra-acetic acid (Trypsin–EDTA, Sigma-Aldrich) and subcultured into a new cell culture flask. The medium was replaced every 2 days. Cells were used for the following experiments.

### 2.3 Cell viability

The 3-(4,5-dimethylthiazol-2-yl)−5-(3-carboxymethoxyphenyl)−2-(4-sulfophenyl)−2 H-tetrazolium (MTS) assay to evaluate cell viability was performed by using CellTiter 96^®^ AQueous One Solution Cell Proliferation Assay (Promega Italia s.r.l., Milan, Italy), following the manufacturer’s protocol, as previously reported (28–32). To evaluate cell growth, in preliminary experiments (data not shown), cells were seeded and incubated at 37°C in a humidified atmosphere (95%) under 5% CO_2_ at different densities for each well (0–500–1,000–2,000–5,000–10,000–20,000–50,000) in 96-well plates. After 24, 48, 72, and 96 h, the plates were read with a SynergyH1 BioTek spectrophotometer (Agilent, Santa Clara, United States) at 490 nm. The cell density of 3,000 cells/well was chosen for cell viability assessment. In brief, cells were seeded at 3,000 cells/well and incubated overnight. Then, cells were treated with six different concentrations of PS-NPs (10–25–50–100–250–500 μg/mL). Cell viability was assessed after 24, 48, and 96 h of exposure. Negative control wells contained cells unexposed to PS-NPs but treated with MTS reagent. The culture medium alone or the culture medium including PS-NPs at tested concentrations was used as a control to determine the background values. After 3 h of incubation of the cells with the reagent in the dark, the plates were read at 490 nm with a SynergyH1 BioTek spectrophotometer (Agilent). The test was performed by evaluating two biological replicates and three technical replicates for each condition.

### 2.4 ROS production

The ROS production was evaluated using the ROS-GloTM H_2_O_2_ Assay kit following the manufacturer’s recommendations (33). In brief, 3.000 cells/well were seeded in white opaque 96-well plates (Corning, Sigma-Aldrich) and left to adhere overnight. Then, H_2_O_2_ substrate solution was added to the wells, and cells were treated with 6 different concentrations of PS-NPs (10–25–50–100–250–500 μg/mL) for 24, 48, and 96 h. Two hours before the end of the treatment, 100 μL of ROS-Glo detection solution was added to each well. After incubation at room temperature for 20 min, the luminescence was measured with a GloMax^®^ Multi Detection System luminometer (Agilent, Santa Clara, United States). Two hours of treatment with menadione (20 μM) was carried out as a positive control, which produced a concentration of H_2_O_2_ of approximately 76 μM (data not shown). A standard curve with H_2_O_2_ concentrations in the 0–100 μM range was constructed. The experiments were conducted in wells containing only medium or with seeded cells. The test was performed using six independent experiments for each condition.

### 2.5 Quantification of IL-8/CXCL8 levels

A549 cells (500,000/well) were treated for 48 h with PS-NPs (10, 100, and 500 μg/mL). Then, cells were recovered for RNA extraction, as described below, while supernatants were collected and centrifuged at 14,000 rpm for 5 min to analyze protein levels of IL-8/CXCL8 by using the Human IL-8/CXCL8 Quantikine ELISA Kit (R&D System, Bio-Techne), according to the manufacturer’s procedure.

### 2.6 RNA extraction and qRT-PCR

Total RNA was extracted from A549 cells using a Pure link RNA Mini kit according to the manufacturer’s protocols. After removal of the genomic DNA through the DNAse kit, total RNA (2 μg) was reverse transcribed into cDNA using Iscript cDNA Synthesis Kit (Bio-Rad, Milan, Italy) according to the manufacturer’s protocols. For the reaction mixture, 100 ng of cDNA was used. For the assessment of gene expression in A549 cells, the amplification of GAPDH (protein name: glyceraldehyde-3-phosphate dehydrogenase), SOD1 (protein name: superoxide dismutase 1, soluble), SOD2 (protein name: superoxide dismutase 2, mitochondrial), CAT (protein name: catalase), GPX1 (protein name: glutathione peroxidase 1), HMOX1 (protein name: heme oxygenase 1), CDKN1A (protein name: cyclin dependent kinase inhibitor 1A), IL1A (protein name: interleukin 1 alpha), IL1B (protein name: interleukin 1 beta), IL6 (protein name: interleukin 6), CXCl8 (protein name: C-X-C motif chemokine ligand 8), BCL2 (protein name: BCL2, apoptosis regulator), BAX (protein name: BCL2 associated X, apoptosis regulator), and CASP3 (protein name: caspase 3) was performed using TaqMan gene expression assays (Hs99999905, Hs00533490, Hs00167309, Hs00156308, Hs00829989, Hs01110250, Hs00355782, Hs00174092, Hs01555410, Hs00174131, Hs00174103, Hs04986394, Hs00180269, and Hs00234387, respectively) according to the manufacturer’s instructions. Genes mRNA levels were normalized to Gapdh levels using a 7900HT Real-Time PCR (Applied Biosystems, CA, United States).

### 2.7 Senescence detection

The *β*-gal Substrate 4-Methylumbelliferyl *β*-d-galactopyranoside (4-MUG) consumption was used to detect Senescence-associated beta-galactosidase (SA-*β*-gal) activity in A549 cellular lysates. The cells were treated with 10, 100, and 500 μg/mL PS-NPs for 96 h. Upon binding to *β*-gal, 4-MUG is hydrolyzed to the fluorescent product 4-MU, which is measured at an excitation wavelength of 360 nm and an emission wavelength of 465 nm. Fluorescent intensity correlates with *β*-gal levels present in the sample. The experiment was performed with a cellular senescence assay kit (CellA28 Biolabs, CBA 231) according to manufacturer instructions. The value of each SA-*β*-Gal activity was standardized by its total protein concentration. Each data was measured from triple independent experiments.

### 2.8 Assessment of protein levels by Western blot analysis

A549 cells (500,000/well), untreated or treated with 10, 100, and 500 μg/mL PS-NPs for 48 h, were lysed in PBS Triton 1% with 1 mM of phenylmethylsulfonyl fluoride (PMSF) and complete ethylenediaminetetraacetic acid (EDTA)-free proteases inhibitors (Thermo Scientific, Rockford, IL United States). Proteins were loaded onto 4–15% Sodium Dodecyl Sulfate-PolyAcrylamide Gel Electrophoresis (SDS-PAGE), transferred to Polyvinylidene Difluoride (PVDF) membranes (GE Healthcare, Milan, Italy), and incubated with anti-Caspase 3 (Cell Signaling Technology, Danvers, MA), anti-Bcl-2 (Abnova Corporation, Taipei City, Taiwan), anti-p21 (Santa Cruz Biotechnology, Santa Cruz, CA, United States), and anti-*β*-actin (Cell Signaling Technology, Danvers, MA) antibodies. Membranes were developed using ECL Western Blotting Detection Reagents and analyzed using Alliance 1 D software (UVITEC, Cambridge, United Kingdom). The quantification of optical density (OD) of different specific bands was calculated and normalized to the OD of *β*-actin.

### 2.9 Flow cytometry analyses

To assess apoptosis, a FITC Annexin-V apoptosis detection kit (BD Pharmingen, San Diego, CA, United States) was used following the manufacturer’s instructions. The day before PS-NP treatment, cells were seeded at a density of 500,000/well and grown under standard culture conditions in 6-well plates. After 24 h, 10, 100, and 500 μg/mL of PS-NPs were added to cells, which were further incubated. After 96 h, 10^5^ cells were gently re-suspended in 100 μL of binding buffer and incubated for 15 min at room temperature in the dark with 5 μL of Annexin-V-FITC and 5 μL of Propidium Iodide (PI). After the addition of 200 μL of binding buffer, samples were analyzed with a Cytoflex flow cytometer with the FL1 and FL3 detector in a log mode, using the Cytoexpert analysis software (both from Beckmann Coulter, Milan, Italy). For each sample, at least 5,000 events were collected. From the same samples, the dot plots of the autofluorescence parameters (forward and side scatter) were analyzed for changes in cell size and cellular content.

### 2.10 Dynamic light scattering (DLS)

The hydrodynamic diameter of PS-NPs was evaluated by dynamic light scattering (DLS, Anton Paar Litesizer 500 Graz, Austria). PS-NPs were diluted 1:40, based on the optimized parameters such as the mean intensity (kcount/s) and the filter OD, considering that the PS-NPs detectable concentration was 400-fold lower than the considered dilution factor. The samples were thermostated for 2 min at 25°C before measurements, which were performed in a 2.5-mL disposable cuvette and then analyzed in triplicate using a 175° detection angle. The autocorrelation functions were fitted by applying the Kalliope software (Anton Paar, Graz, Austria).

### 2.11 Transmission electron microscopy (TEM)

The cells were washed twice in PBS at 37°C, fixed in 3.5% glutaraldehyde in 0.1 M sodium cacodylate buffer, pH 7.2, and kept in fixative at 4°C before further use. For thin sectioning, the cells were post-fixed in 2% OsO4 for 2 h at room temperature and then contrasted in saturated uranyl acetate for 2 h at room temperature. The samples were embedded in Epon 812. EM ultra-thin sections (~50 nm of thickness) were cut from embedded samples using a Leica Ultracut R microtome (Leica Microsystem, Wien, Austria) with a 45° Diatome Ultra diamond knife (Diatome, Biel, Switzerland) and stained with lead citrate. Sections were viewed and photographed in a 120 kV JEM-1400 Flash Transmission Electron Microscope (TEM) (Jeol Ltd., Tokyo, Japan) equipped with CMOS camera Matataki and TEM Center software (Jeol Ltd.) at a magnification ranging between 2,500 and 50,000×.

### 2.12 Statistical analysis

The data were statistically analyzed using the Student’s *t*-test, and the values of the treated samples were compared with those of the untreated ones. All quantitative results were analyzed using Graph Pad Prism Software (version 9.00 for Mac; GraphPad, San Diego, CA, United States). Data are presented as means ± SD, unless otherwise specified, of at least three separate experiments, each analyzed at least in duplicate. Values of *p* ≤ 0.05 were considered statistically significant.

## 3 Results

### 3.1 Exposure to PS-NPs affects cell viability

**Figure 1:**
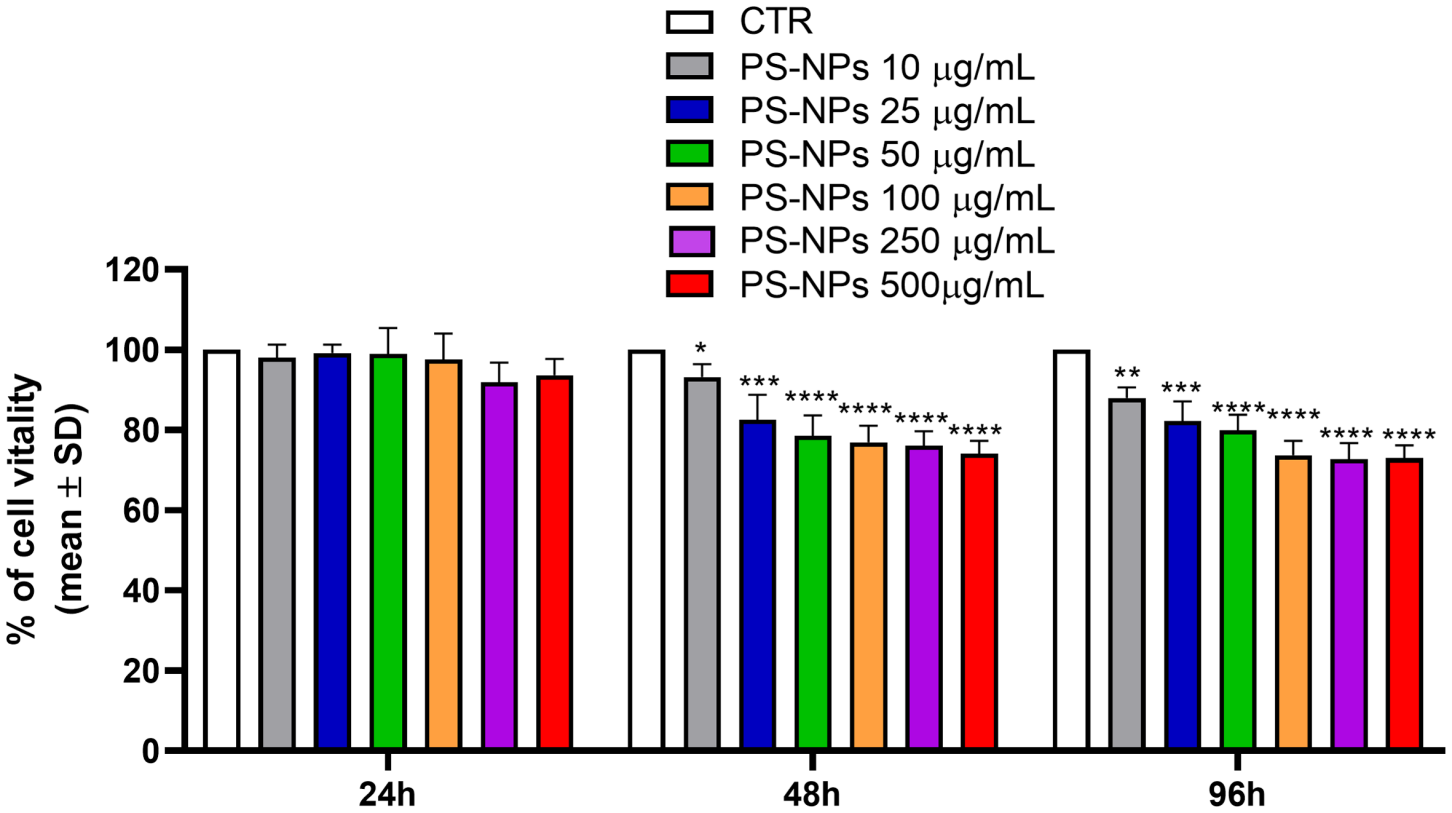
Effects of PS-NPs on A549 cell viability. Increasing concentrations of PS-NPs (10–500 μg/mL) were added to A549 cells (3,000 cells/well). MTS assay was performed after 24, 48, and 96 h of incubation. Each value represents the mean ± SD of six independent experiments. *p-*values are expressed as ^****^p ≤ 0.0001, ^***^p ≤ 0.001, ^**^p ≤ 0.01, and ^*^p ≤ 0.05 vs. untreated cells (CTR).

The DLS analysis confirmed that PS-NPs had a hydrodynamic diameter of 802.0 nm (Supplementary Figure S1). Then, an MTS assay was performed to assess their effect on A549 cell viability. The cells were treated with 10, 25, 50, 100, 250, and 500 μg/mL PS-NPs for 24, 48, and 96 h. At all the concentrations tested, A549 cells treated for 24 h showed no reduction in viability relative to the untreated controls (Figure 1), whereas when exposed for 48 and 96 h, a significant reduction of viability was found at all the concentrations tested, when compared with the control condition. The maximum effect was recorded after treatment with 500 μg/mL PS-NPs for 48 and 96 h, when A549 viability was reduced by 21 and 22%, respectively (Figure 1).

### 3.2 Exposure to PS-NPs induces oxidative stress

**Figure 2:**
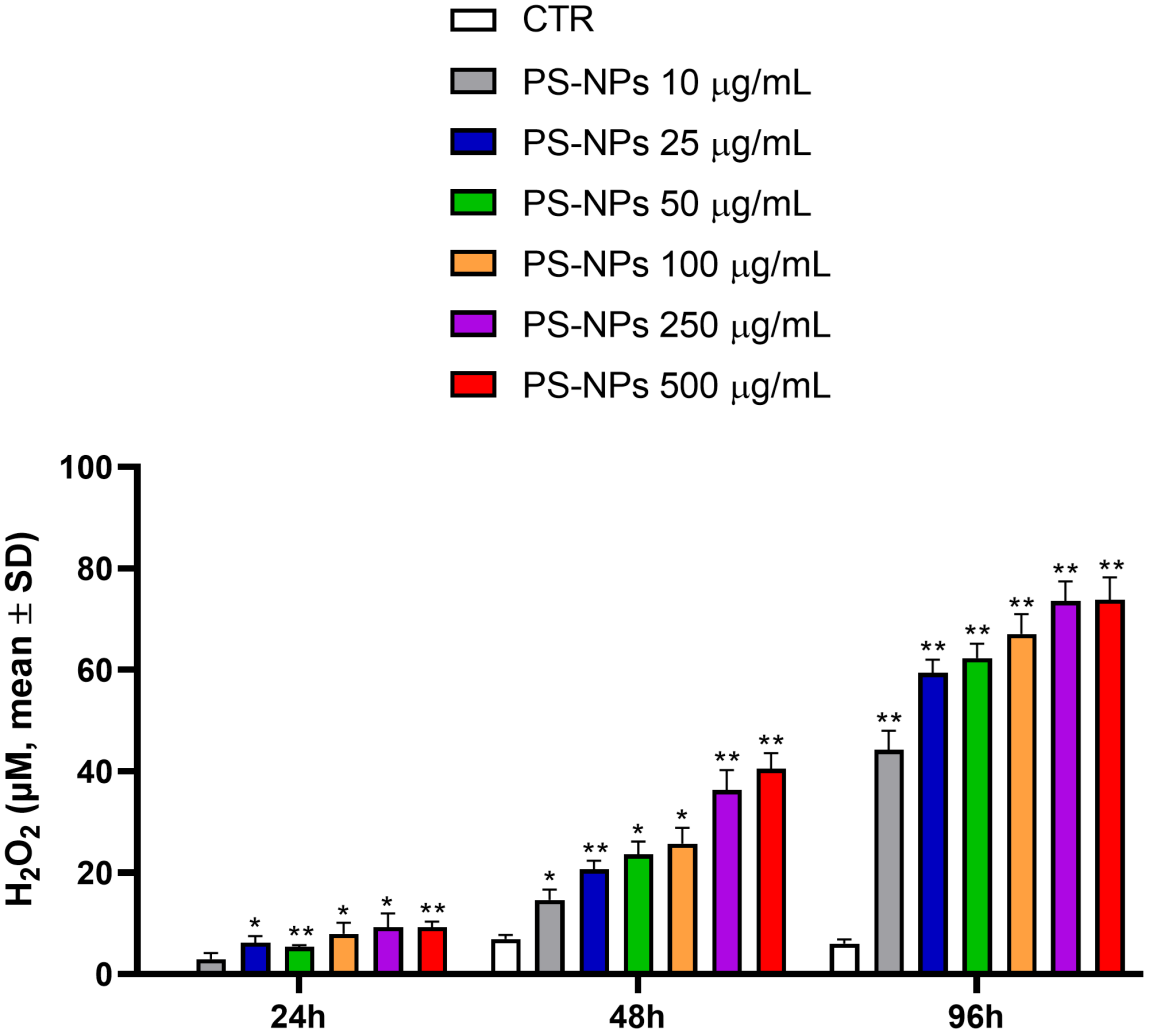
Effects of PS-NPs on the intracellular generation of reactive oxygen species in A549 cells. Increasing concentrations of PS-NPs (10–500 μg/mL) were added to A549 cells (3,000 cells/well). ROS-GloTM H_2_O_2_ assay was performed after 24, 48, and 96 h of incubation. Each value represents the mean ± SD of six independent experiments. *p*-values are expressed as ^**^p ≤ 0.01 and ^*^p ≤ 0.05 vs. untreated cells (CTR).

A rapid and sensitive luminescent assay was used to obtain information about the capability of PS-NPs to induce oxidative stress in A549 cells after 24, 48, and 96 h of treatment. The assay directly measures the hydrogen peroxide (H_2_O_2_) level in the culture medium. This reactive compound is the main ROS species produced by cells and is characterized by the most extended half-life (33). As shown in Figure 2, a significant H_2_O_2_ formation was observed at all the PS-NP concentrations tested in a dose and time-dependent manner. The cell exposure to PS-NPs for 96 h strongly stimulated H_2_O_2_ formation, which reached a maximum of 73.85 μM following treatment with 250 and 500 μg/mL PS-NPs (Figure 2).

Free radicals are potentially toxic; they are usually inactivated or scavenged by antioxidants before they can cause damage to lipids, proteins, or nucleic acids. To ameliorate and cope with injury from oxidative damage and maintain redox homeostasis, eukaryotic cells have a complex defense system that includes antioxidant enzymes, such as SOD, which reduces the superoxide anion into hydrogen peroxide, CAT, and GPX1, that can reduce H_2_O_2_ to H_2_O (34).

Considering the effects of PS-NPs on intracellular H_2_O_2_ production, we analyzed by qPCR the expression levels of SOD1 (encoding for the cytoplasmic isoform) and SOD2 (encoding for the mitochondrial isoform), following A549 cell exposure to 10, 100, and 500 μg/mL of PS-NPs. SOD is considered a crucial player in scavenging most of the superoxide produced by respiration (35, 36). We also analyzed the expression levels of CAT and GPX1 in the same experimental conditions.

**Figure 3:**
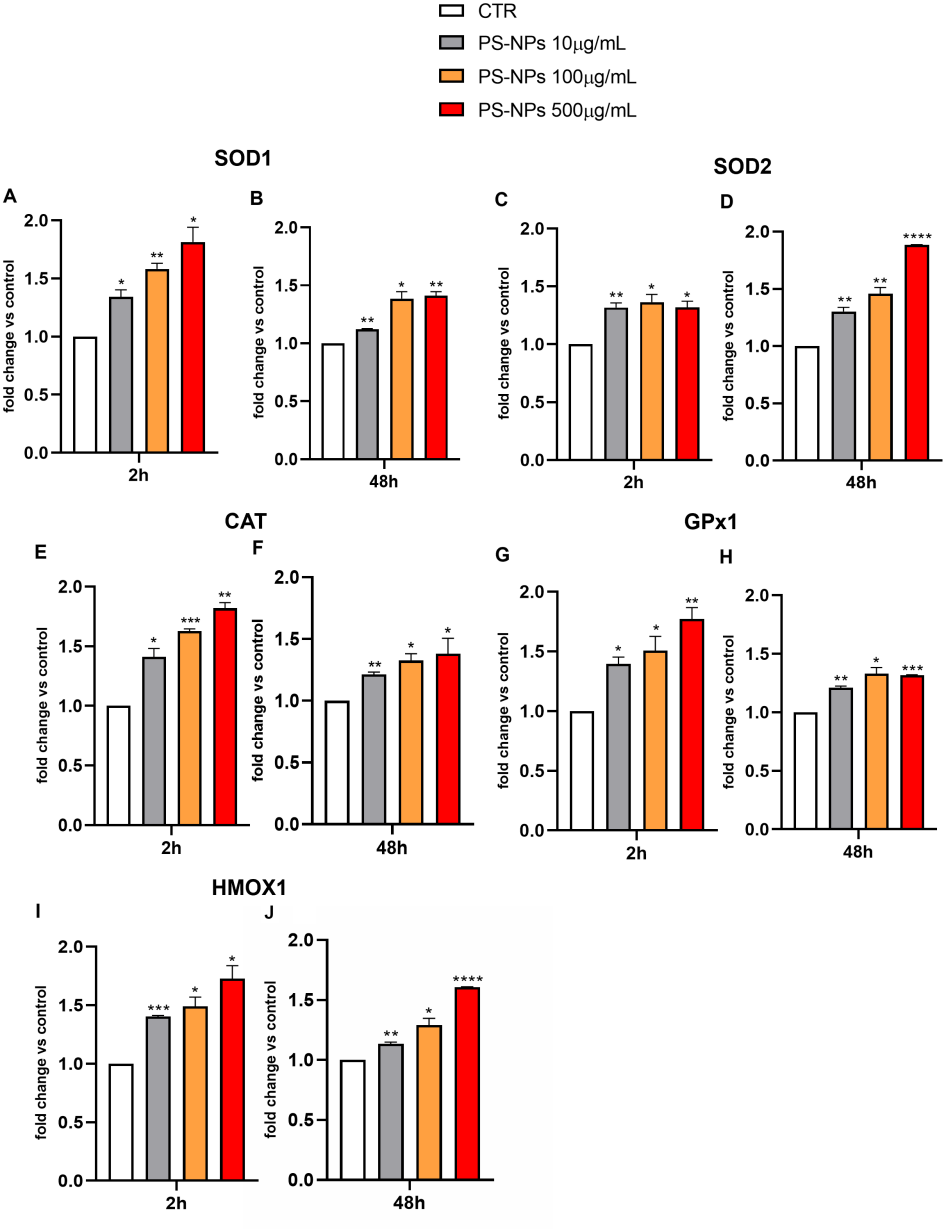
Effects of PS-NPs on the expression of oxidative stress-related genes in A549 cells. Selected concentrations of PS-NPs (10, 100, and 500 μg/mL) were added to A549 cells (500,000/well). After 2 **(A,C,E,G,I)** and 48 h **(B,D,F,H,J)** of incubation, the mRNA levels of SOD1 (protein name: superoxide dismutase 1) **(A,B)**, SOD2 (protein name: superoxide dismutase 2) **(C,D)**, CAT (protein name: catalase) **(E,F)**, GPX1 (protein name: Glutathione Peroxidase 1) **(G,H)**, HMOX1 (protein name: Heme Oxygenase 1) **(I,J)**, and Gapdh were assessed by qPCR. Relative fold change in gene expression was calculated using untreated A549 cells as control (CTR, fold change = 1) and normalized to GAPDH as a housekeeping gene. Data are reported as mean ± SD of at least three independent experiments; *p*-values are expressed as ^****^p ≤ 0.0001, ^***^p ≤ 0.001, ^**^p ≤ 0.01, and ^*^p ≤ 0.05 vs. Control (CTR).

As shown in Figure 3, significant induction of SOD1 was detected as early as 2 h after treatment with all the PS-NP concentrations used (Figure 3A). The expression levels of this gene remained constant after 48 h of cell exposure to the stimuli (Figure 3B). Similar results were observed for the expression levels of SOD2 (Figures 3C,D), CAT (Figures 3E,F), and GPX1 (Figures 3G,H).

Heme oxygenase-1 (HO-1) (encoded by the HMOX1 gene) is another key antioxidant enzyme reported to be highly expressed in the lungs (37). It catabolizes heme into ferrous iron, carbon monoxide, and biliverdin/bilirubin and represents the only inducible isoform within the HO family, which comprises two further constitutive forms, HO-2 and HO-3 (38). Similar to SOD1, SOD2, CAT, and GPX1, PS-NPs were able to significantly induce the HMOX1 expression at all tested concentrations (Figures 3I,J).

### 3.3 Exposure to PS-NPs induces cellular senescence

Together with apoptosis, cellular senescence is recognized as an equally important although biologically different type of ultimate cell-cycle exit program because of its lastingly persistent nature and cell-intrinsic and extrinsic roles within the tissue (39).

**Figure 4:**
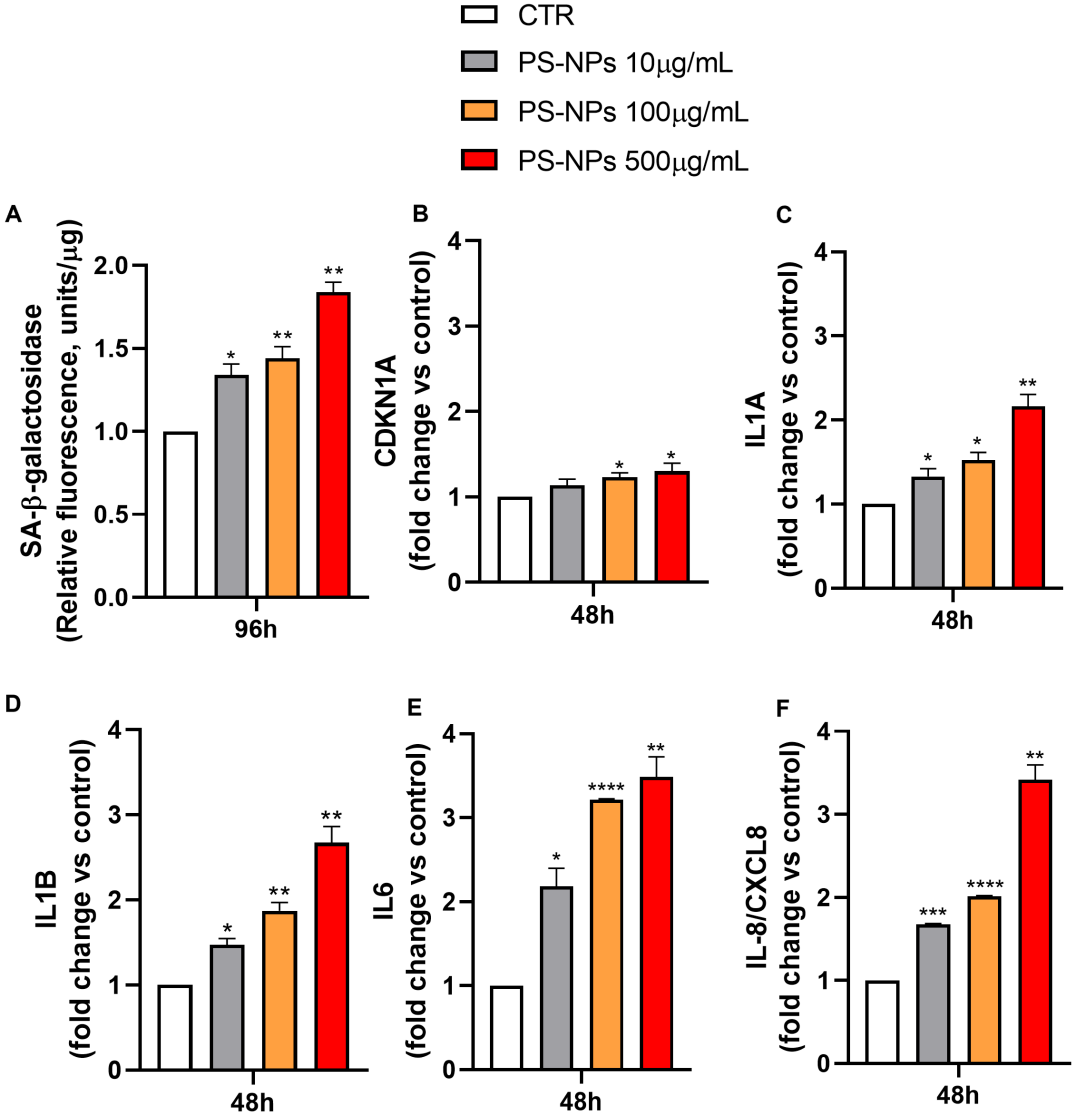
Effects of PS-NPs on cellular senescence induction. Selected concentrations of PS-NPs (10, 100, and 500 μg/mL) were added to A549 cells (500,000/well) to evaluate effects of PS-NPs on SA-β-Gal activity **(A)** and the mRNA levels of CDKN1A **(B)**, IL1A **(C)**, IL1B **(D)**, IL6 **(E)**, and IL-8/CXCL8 **(F)** in A549 cells. The senescence-associated SA-β-Gal activity was evaluated after 96 h of incubation. Data are reported as mean ± SD of three independent experiments **(A)**. The mRNA levels of CDKN1A (protein name: Cyclin-Dependent Kinase Inhibitor 1A, also called p21), IL1A (protein name: Interleukin 1 Alpha), IL1B (protein name: Interleukin 1 beta), IL6 (protein name: Interleukin 6), IL-8/CXCL8 (protein name: Interleukin 8), and GAPDH were assessed by qPCR after 48 h of incubation. Relative fold change in gene expression was calculated using untreated A549 cells as control (CTR, fold change = 1) and normalized to GAPDH as a housekeeping gene. Data are reported as mean ± SD of at least three independent experiments; p-values are expressed as ^****^p ≤ 0.0001, ^***^p ≤ 0.001, ^**^p ≤ 0.01, and ^*^p ≤ 0.05 vs. control (CTR).

In addition to the proliferation arrest, other typical features characterize senescent cells, including the senescence-associated β-galactosidase activity (SA-*β*-Gal) and the secretion of a vast spectrum of cytokines, chemokines, proteases, and growth factors, referred to as senescence-associated secretory phenotype (SASP) (40, 41). To assess the potential capability of PS-NPs in inducing senescence, the SA-β-Gal activity in A549 cells exposed to these pollutants was evaluated. As shown in Figure 4A, PS-NPs were able to significantly induce SA-*β*-Gal activity, measured in A549 cell lysates, at all the concentrations tested. Five hundred μg/mL PS-NPs caused an increase of SA-β-Gal activity by 1.84-fold when compared to control cells (*p* ≤ 0.01).

To further confirm the senescence occurring after 96 h of PS-NP exposure, we evaluated, by qPCR, the earlier mRNA expression levels of key cell molecules reported as markers of senescence, including CDKN1A, encoding for the protein Cyclin-Dependent Kinase Inhibitor 1A, also called p21, IL1A, IL1B, IL6, and IL-8/CXCL8, encoding for cytokines belonging to the SASP family (Figures 4B–F).

After 48 h of incubation, CDKN1A expression was increased by 13, 23, and 30% following cell exposure to 10, 100, and 500 μg/mL PS-NPs, respectively (Figure 4B). The induction of p21 was also confirmed by Western blot analysis in A549 cells treated for 48 h with PS-NPs (Supplementary Figures S2, S3). In the same way, but with a higher magnitude, IL1A, IL1B, IL6, and IL-8/CXCL8 mRNA levels were augmented when the cells were treated with the same concentrations of PS-NPs, and the effect is dose-dependent (Figures 4C–F).

Within the molecules analyzed as SASP, IL-8/CXCL8 is known to exert a crucial role in lung inflammation *in vivo*. It is also involved in modulating the hyperinflammatory response present in acute respiratory distress syndrome (ARDS) (42). Moreover, IL-8/CXCL8 levels have been reported to be dramatically higher in non-survivors compared to survivors of people with COVID-19 (43). Thus, we also evaluated the release of IL-8/CXCL8 in A549 cells in basal conditions and after exposure to PS-NPs (10, 100, and 500 μg/mL) for 48 h. The results showed a marked increase in IL-8/CXCL8 protein levels present in the cell medium at all the PS-NP concentrations used, thus confirming the upregulation described at the transcriptional level (Supplementary Figure S4).

### 3.4 Exposure to PS-NPs induces cellular apoptosis

**Figure 5:**
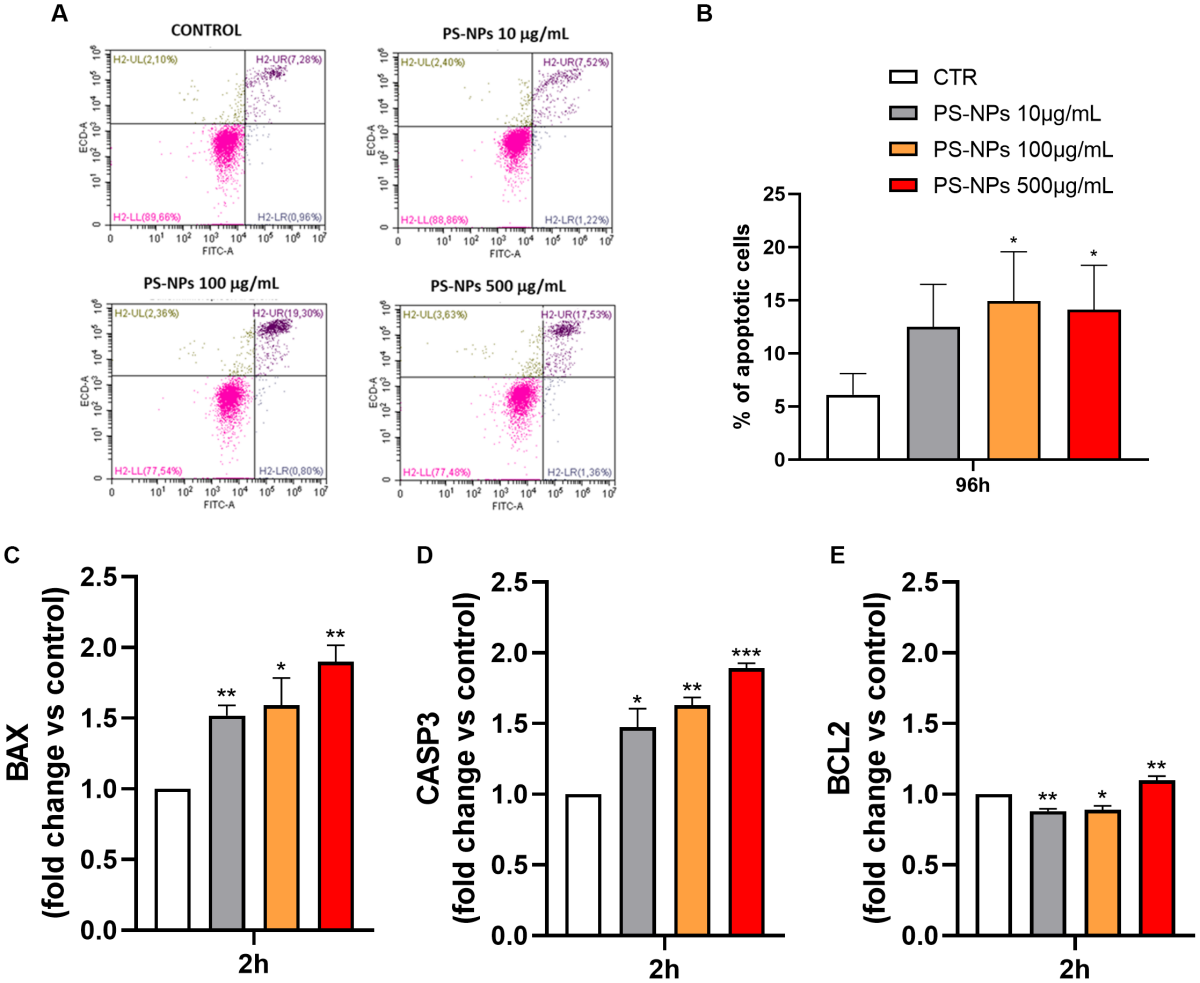
Effect of PS-NPs on apoptosis in A549 cells. Selected concentrations of PS-NPs (10, 100, and 500 μg/mL) were added to A549 cells (500,000/well). Apoptosis was measured by Annexin-FITC/PI assay by flow cytometry after 96 h of incubation. Apoptotic cell population (Annexin-Vpos/PIneg and Annexin-Vpos/PIpos) can be discriminated from vital (Annexin-Vneg/PIneg) or necrotic cells (AnnexinVneg/PIpos), according to their fluorescence emission. **(A)** Dot plots are the most representative of the four experiments. **(B)** The graph represents the mean percentage ± SD of four experiments. Control (CTR) corresponds to untreated A549 cells. *p*-values are expressed as ^*^p ≤ 0.05 vs. control (CTR). The mRNA levels of BAX **(C)**, CASP3 **(D)**, BCL2 **(E)**, and GAPDH were assessed by qPCR after 2 h of incubation. Relative fold change in gene expression was calculated using untreated A549 cells as control (CTR, fold change = 1) and normalized to GAPDH as a housekeeping gene. Data are reported as mean ± SD of at least three independent experiments; *p*-values are expressed as ^***^p ≤ 0.001, ^**^p ≤ 0.01, and ^*^p ≤ 0.05 vs. control (CTR).

Once the senescence occurrence was assessed, the apoptotic pathway was investigated as a potential alternative defense mechanism of A549 cells against the PS-NPs. The same three concentrations (10, 100, and 500 μg/mL) were chosen, and a flow cytometry Annexin-V/PI assay was performed after 96 h of incubation. Figures 5A,B show an increase in the percentage of apoptotic cells in all the samples exposed to the PS-NPs, with the 100 and 500 mg/mL having about a threefold increase when compared to the untreated cells.

In Figures 5C–E, changes in the expression of the three apoptosis-related genes BAX, CASP3, and BCL2 are shown. Interestingly, the expression levels of the pro-apoptotic genes BAX (Figure 5C) and CASP3 (Figure 5D) were found to increase after just 2 h of exposure to the plastics, which is in line with the previous results. On the other hand, the anti-apoptotic gene BCL2 decreased at the lowest PS-NP concentrations, 10 and 100 μg/mL, and showed a slight increase at the highest concentration of 500 μg/mL (Figure 5E). As shown in Supplementary Figures S5, S6, in A549 cells incubated with PS-NPs for 48 h, the protein levels of Caspase 3 decreased (Supplementary Figures S5A,B), while the levels of fragmented protein were detectable only in PS-NPs-treated samples, suggesting the activation of apoptosis cascade (Supplementary Figures S5A,C). At the same time point, Bcl-2 protein expression was upregulated in treated cells (Supplementary Figures S5D,E).

Interestingly, flow cytometry dot plots for physical parameters (forward scatter vs. side scatter) showed an increase in the side scatter of all the cells, already at the concentration of 100 μg/mL, and further increased when 500 μg/mL of PS-NPs were administered to the cells (Supplementary Figure S7).

### 3.5 Cellular uptake of PS-NPs

Since the side scatter indicates the internal complexity, i.e., the granularity of the cells, we studied the internalization of PS-NPs in A549 cells by TEM, confirming the internalization by target cells.

**Figure 6:**
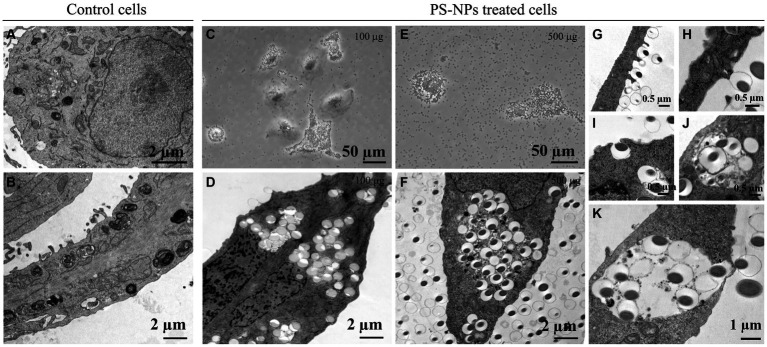
Cellular intake of PS-NPs in A549 cells after 100 μg/mL or 500 μg/mL treatment. Transmission electron microscopy (TEM) analysis revealed the movement of nano-sized plastics into membrane structures **(A,B,D,F,G–K)**. Active transport through the cell membrane (insets in **G–K**) may represent a significant gateway into the cell after treatment with different PS-NP concentrations (100 and 500 μg). Panels **(C,E)** reported images acquired from optical microscopy. Bars: **(A,B,D,F)** 2 μm; **(C,E)** 50 μm; **(G–J)** 0.5 μm; **(K)** 1 μm.

Untreated A549 cells show electron-dense particles and autophagic vacuole (mainly multilamellar bodies), characteristics of adenocarcinoma cells (Figures 6A,B), which was increased in A549 cells treated with PS-NPs 100 μg/mL (Figures 6C,D) or 500 μg/mL (Figures 6E,F) in a concentration-dependent manner. At higher concentrations PS-NPs appear dispersed inside cellular endosomes (circular white areas), and cells start showing signs of toxicity. In Figures 6G–K, different phagocytosis steps are appreciable: (i) a PS-NP is “presented” on the surface of the A549 cell (Figures 6G,H); (ii) formation of phagocytic cups around PS-NP (Figure 6I); (iii) inclusion of several subsequent PS-NPs inside the same cell (Figure 6K), and (iv) process completion, PS-NPs are encapsulated within a phagosome, and start of the autophagic process (Figure 6J).

## 4 Discussion

Nanoplastics, a novel global environmental hazard, have become ubiquitous in daily life and pose a significant health risk when inhaled and lodged in the respiratory system. In our current research, we utilized A549 cells, a human lung adenocarcinoma cell line, and specifically employed PS-NPs as surrogates to explore the respiratory toxicity associated with airborne NPs. The selection of PS-NPs 800 nm was deliberate since this particular size range has been poorly investigated (27), and prior studies have linked distinct toxicities with different particle sizes (21, 44). Moreover, it is well known that developing analytical methodologies to identify and quantify plastic particles in complex matrices is an urgent need for research concerning quantitative toxicity assessment of plastic pollution. In this context, according to recent methodological studies (45), we are developing a flow cytometry analytical method to quantify plastic particles in blood from humans and mice. Preliminary data (not shown) indicated that it will allow detection of PS-NPs 800 nm. This analytical method will be used to perform *in vivo* studies designed to assess the impact of PS-NP (800 nm) exposure on the respiratory system and correlate it with PS-NP circulating levels.

Inhalation doses of plastic particles can vary depending on the type of textiles in use and other indoor environmental factors (such as ventilation) (46), and no standard method exists for determining NP and MP levels in air and dust. According to previous *in vitro* studies on A549 cells (21, 23, 24), we investigated the effects of PS-NPs (800 nm) exposure on this cell line at a concentration of 10–500 μg/mL, ranging from PS-MP concentrations experienced by residents of urban areas to those probably reflecting high-level exposure for workers at industrial manufacturing sites (24).

Our investigation unveiled that PS-NPs markedly decreased cell viability in a concentration-dependent manner. Additionally, these PS-NPs disrupted the cellular redox balance and activated pathways, leading to cellular senescence or apoptosis. This resulted in the production of SASP and eventual cell death.

The internalization of PS particles is a crucial step for cellular toxicity, and several processes involved in the mechanism of internalization have been proposed, depending upon the physiology of particles, such as size, charge, zeta potential, and shape (27). In this study, the internalization of PS-NPs (800 nm) was first suggested by alteration of the internal complexity or granularity (i.e., side scatter) of cells in a concentration-dependent manner (Supplementary Figure S7). TEM microscopy analyses confirmed the uptake of PS-NPs, describing effective internalization by a high level of phagocytosis. This process represents a mechanism of isolation of PS-NP particles aiming to reduce their cellular toxicity but represents a strict concentration-related reaction.

The aforementioned structural and functional analyses indicated that cell death resulting from redox imbalance likely plays a central role in lung injury induced by PS-NPs. Thus, we assessed oxidative stress levels, known for damaging cellular macromolecules—DNA, lipids, and proteins—ultimately leading to lung cell death and tissue damage (34, 47). Our study encompassed various indicators, including the production of hydrogen peroxide and expression of the main hallmark genes included in the antioxidant network, GPX1, CAT, SOD1, and SOD2, to comprehensively gauge the process (48, 49). Additionally, the gene expression of HMOX1, whose product is a ubiquitous stress-response protein, significantly increased following PS-NP treatment, signifying considerable oxidative stress. These changes in gene expression were significant already after 2 h of PS-NP exposure, up to at least 48 h, and were concentration-dependent. However, the expression of SOD2, the manganese-dependent, mitochondrial isoform, reached a maximum induction level already at the exposition to the lower concentration of PS-NPs (i.e., 10 μg/mL), suggesting that cytoplasm could be more interested and sensitive to an exogenous stimulus such as PS-NPs exposure. This collective analysis strongly suggests that PS-NPs induce oxidative stress, leading to lung cell death and tissue damage, potentially contributing to the pathogenesis of lung diseases. The cascade of critical adverse events initiated by the oxidative stress response to PS-NPs underscores its significance (50).

The decline in cell viability and the substantial, concentration-dependent increase in ROS production imply that PS-NP exposure poses potential genotoxic stress to A549 cells, jeopardizing genomic integrity. When the DNA repair machinery fails to mend damaged sites during a temporary cell-cycle arrest or when overwhelming genotoxic stress surpasses the repair capacity, cellular defense mechanisms such as apoptosis or senescence are activated to prevent the uncontrolled proliferation of these damaged and potentially harmful cells.

We established primarily that PS-NPs amplify cell death and tissue injury. Previous studies have confirmed the capacity of PS-MPs to induce apoptosis across various tissues and organs, including reproductive (51, 52), cardiovascular (53), and nervous systems (54). In our study, PS-NPs induced dose-dependent lung cell apoptosis, as revealed by FACS analysis. Further exploration into biological mechanisms unveiled significant activation of various apoptotic pathways in PS-NPs-treated lung cells. The use of PI allowed us to distinguish between apoptotic and necrotic cell death. However, it is crucial to note that nanoparticle-induced cell death encompasses a mixed form, not a singular mode (55). We described that expression levels of the pro-apoptotic gene BAX and the effector gene CASP-3 increase after 2 h of exposure to the plastics, which is in line with the previous results. On the other hand, the expression of the anti-apoptotic gene BCL-2 decreased at the lowest concentrations, namely 10 and 100 μg/mL, and showed a slight increase at the highest concentration, i.e., 500 μg/mL. Future investigations involving pyroptosis, autophagy, and ferroptosis are necessary for a comprehensive understanding of lung cell death induced by PS-NPs.

Despite decades of research primarily focusing on apoptosis, cellular senescence is increasingly acknowledged as an equally vital but fundamentally distinct cell-cycle exit program due to its persistent nature and multifaceted roles within both the tissue and tumor microenvironment. The cellular senescence, regarded as a cellular stress response, may have both beneficial and detrimental effects mediated by released bioactive molecules, including the SASP (39–41). This last has been reported to promote tumorigenesis and induce epithelial-to-mesenchymal transition, thus suggesting a role in metastasis formation (56). Typically, senescent cells are eliminated from the environment by infiltrating immune cells. However, when their clearance becomes compromised, as can happen under chronic exposure to environmental pollutants, they accumulate in tissues (57). Senescent cells exhibit distinct characteristics beyond proliferation arrest, including enlarged cellular morphology, the presence of SA-β-Gal, and the secretion of a diverse array of cytokines, chemokines, proteases, and growth factors constituting the SASP. In our experimental setup, due to the absence of a specific senescence-defining marker, assessing SA-β-Gal activity demonstrated that exposure to 10, 100, and 500 μg/mL of PS-NPs for 96 h induced senescence (Figure 4A). One of the hallmarks of the senescent phenotype, CDKN1A, increased its expression up to 30% following cell exposure to 500 μg/mL PS-NPs (Figure 4B). In the same way, but with a higher magnitude, IL1A, IL1B, IL6, and IL-8/CXCL8 mRNA levels were augmented when the cells were treated with the same concentrations of PS-NPs (Figures 4C–F). These effects followed a concentration-dependent trend (Figure 4). Within the molecules analyzed as SASP, we also evaluated the release of IL-8/CXCL8 protein in A549 cells under basal conditions and after exposure to PS-NPs (10, 100, and 500 μg/mL) for 48 h. The results showed a marked increase in IL-8/CXCL8 protein levels present in the cell medium at all the PS-NP concentrations used, thus confirming the upregulation described at the transcriptional level (Supplementary Figure S4). IL-8/CXCL8 is known to exert a crucial role in lung inflammation *in vivo*. It is also involved in the modulation of the hyperinflammatory response present in ARDS, a devastating pathological condition identified as the cause of 10% of intensive care unit admissions, with up to 23% of patients requiring mechanical ventilation. In ARDS, a role as a biomarker and prognostic factor for this proinflammatory cytokine has been hypothesized (42). Moreover, IL-8/CXCL8 levels have been reported to be dramatically higher in non-survivors compared to survivors of people with COVID-19 (43).

In conclusion, our study supports the emerging evidence indicating the deleterious effects of PS-NPs on lung epithelial cells (A549). At the tested size (800 nm), PS-NPs can enter lung epithelial cells, triggering oxidative stress, senescence, and cell death. Furthermore, these airborne particles may incite a senescent phenotype, elevating basal inflammation levels and diminishing lung tissue repair capabilities, potentially leading to prolonged lung diseases following chronic exposure.

Research on the respiratory toxicity of MPs/NPs remains significantly limited (20, 21, 23). A limitation of our study is the use of submerged cultures. Our future research will prioritize the comparison of the cytotoxicity of PS-NPs, varying their chemical composition and sizes in lung cells using a more advanced *in vitro* model in which epithelial lung cells are grown at the air-liquid interface (ALI) (58), thus better mimicking *in vivo* conditions.

Microplastics and nanoplastics pervade the air, posing a significant threat to human health. While occupational studies highlight pulmonary effects linked to PS-NP inhalation, the tested concentrations likely surpass actual environmental levels. Notably, occupational particles might exhibit lower sorption of co-pollutants such as transition metals, organic compounds, and pathogenic microorganisms than environmental particles. Many adverse health effects associated with NPs might result from the desorption of these contaminants within the respiratory system post-inhalation. An essential inquiry pertains to whether inhaled plastics can translocate to the bloodstream or migrate to mediastinal lymph nodes. Given the diverse characteristics, sources, and fates of plastics, there is a need to reconceptualize MPs/NPs as distinct contaminant classes rather than singular pollutants—a viewpoint echoed by Rochman et al. (59). Recognizing the substantial threat to human health posed by inhaled NPs is crucial.

PS-NPs induce specific effects on the cellular phenotype by induction of oxidative stress and inflammation. In particular, we highlight that in addition to apoptosis induction, PS-NPs increase cellular senescence in a model of alveolar epithelial cells. This is the basis for a deeper understanding of the health effects of a “new” and partly unknown toxicological agent. The induction of cellular senescence is part of a broader framework of effects, which provides links with the pathophysiology of various chronic age-related diseases and tumors and, at the same time, allows to hypothesize scenarios for the design of new epidemiological studies.

## Data availability statement

The raw data supporting the conclusions of this article will be made available by the authors, without undue reservation.

## Author contributions

CM: Data curation, Investigation, Writing – original draft. EA: Data curation, Writing – review & editing, Formal analysis. PDC: Conceptualization, Writing – review & editing. AP: Conceptualization, Writing – review & editing. MG: Formal analysis, Investigation, Writing – review & editing. AB: Data curation, Writing – review & editing. MD: Investigation, Data curation. LM: Investigation, Writing – review & editing. MPD: Formal analysis, Writing – review & editing. VDG: Data curation, Writing – original draft. CP: Formal analysis, Writing – original draft. MP: Conceptualization, Data curation, Writing – original draft, Writing – review & editing. PB: Conceptualization, Data curation, Writing – review & editing.
