# Supplementary material for: Polystyrene nanoplastics mediate oxidative stress, senescence, and apoptosis in a human alveolar epithelial cell line
Source: Front Public Health. 2024 May 10;12:1385387. doi: 10.3389/fpubh.2024.1385387 (PMC11116779; doi:10.3389/fpubh.2024.1385387)
Supplement: Supplementary file 1 [file Data_Sheet_1.pdf]

Supplementary Material

Polystyrene nanoplastics mediate oxidative stress, senescence, and apoptosis in a human alveolar epithelial cell line

Cristina Milillo, Eleonora Aruffo, Piero Di Carlo, Antonia Patruno, Marco Gatta, Annalisa Bruno, Melania Dovizio, Lisa Marinelli, Marilisa Pia Dimmito, Viviana Di Giacomo, Cecilia Paolini, Mirko Pesce, Patrizia Ballerini

Particle size distribution (intensity)

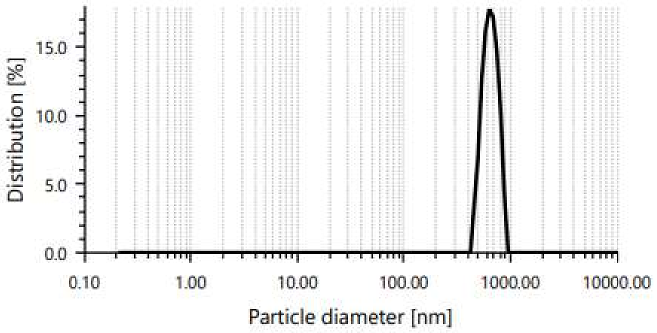

Results

**Hydrodynamic diameter**	802.0 nm	**Mean intensity**	302.8 kcounts/s
**Polydispersity index**	12.3 %	**Absolute intensity**	3399608.0 kcounts/s
**Diffusion coefficient**	0.6 µm²/s	**Intercept g1²**	0.8521

Supplementary Figure 1. Hydrodynamic diameter of PS-NPs.

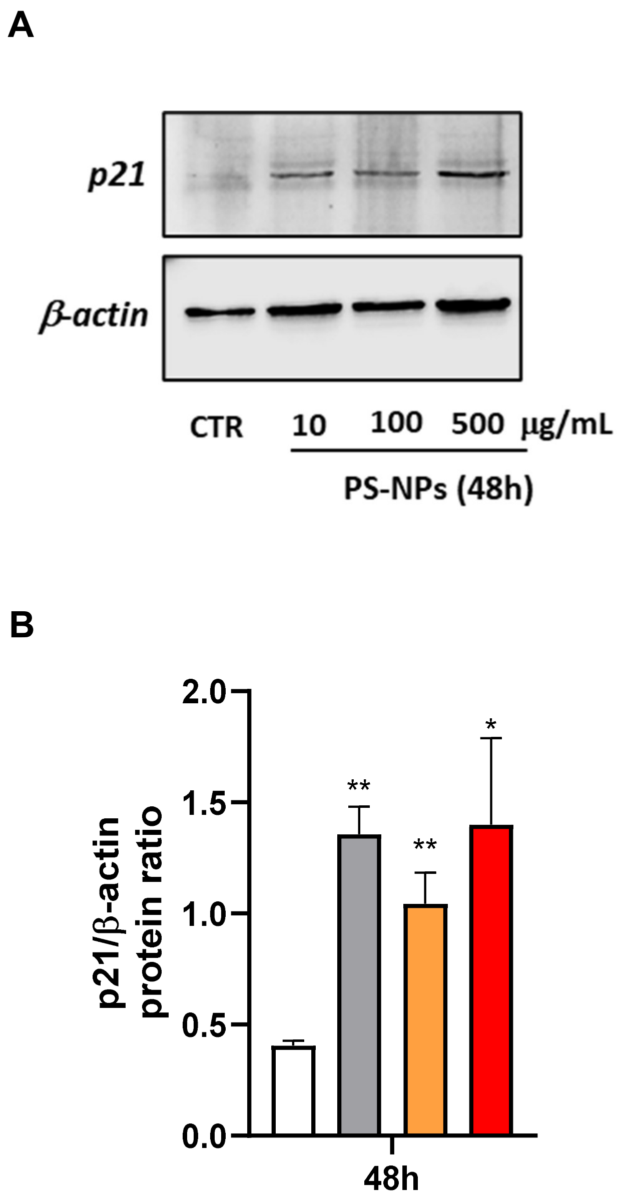

Supplementary Figure 2. (A) Western blot analysis of p21 protein levels in A549 cells (500.000/well) untreated (CTR) or treated with PS-NPs (10, 100 and 500 µg/ml) for 48h. p21 expression level was normalized to β-actin protein expression. (B) Data are reported as ratio between optical density values of p21 immunoreactive bands to that of β-actin bands, as mean ± SD of at least three independent experiments; P values are expressed as *P≤0.05 or ** P≤0.01 vs. CTR.

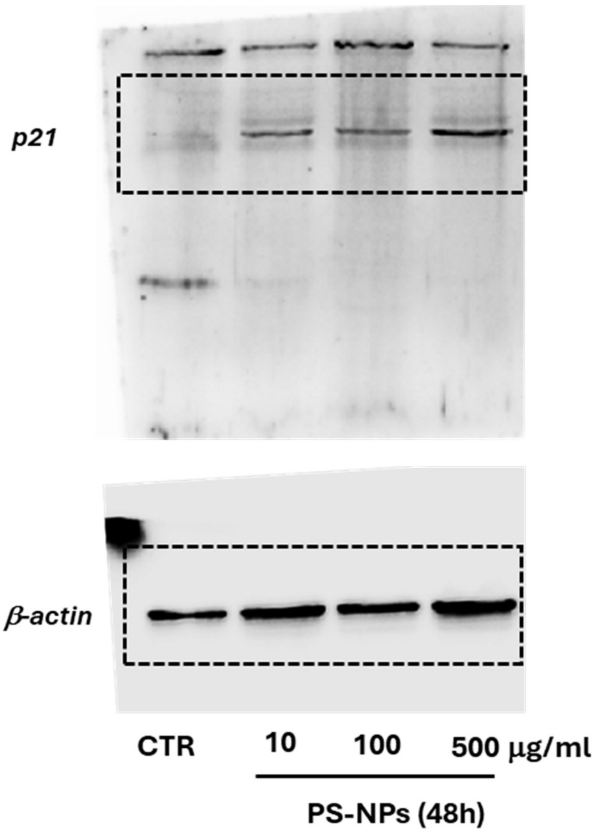

Supplementary Figure 3. The uncropped gel of Supplementary figure 2

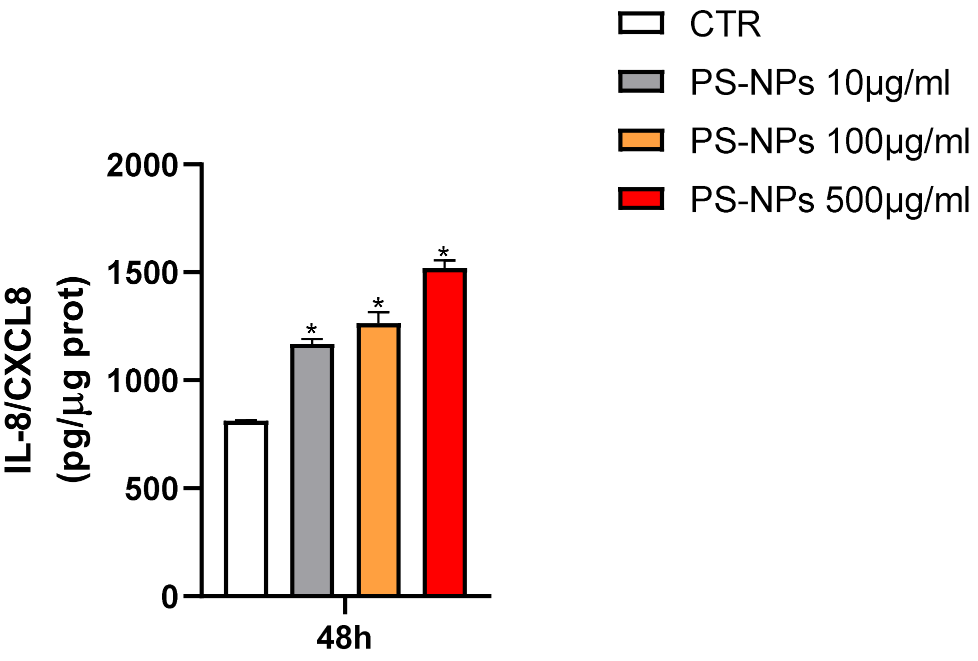

Supplementary Figure 4. Selected concentrations of PS-NPs (10, 100 and 500 µg/ml) were added to A549 cells (500.000/well). The protein levels of IL-8/CXCL8 in cultured media were assessed by ELISA 48 h of incubation. Data are reported as mean ± SD of at least three independent experiments; P values are expressed as *P≤0.05 vs. Control (CTR).

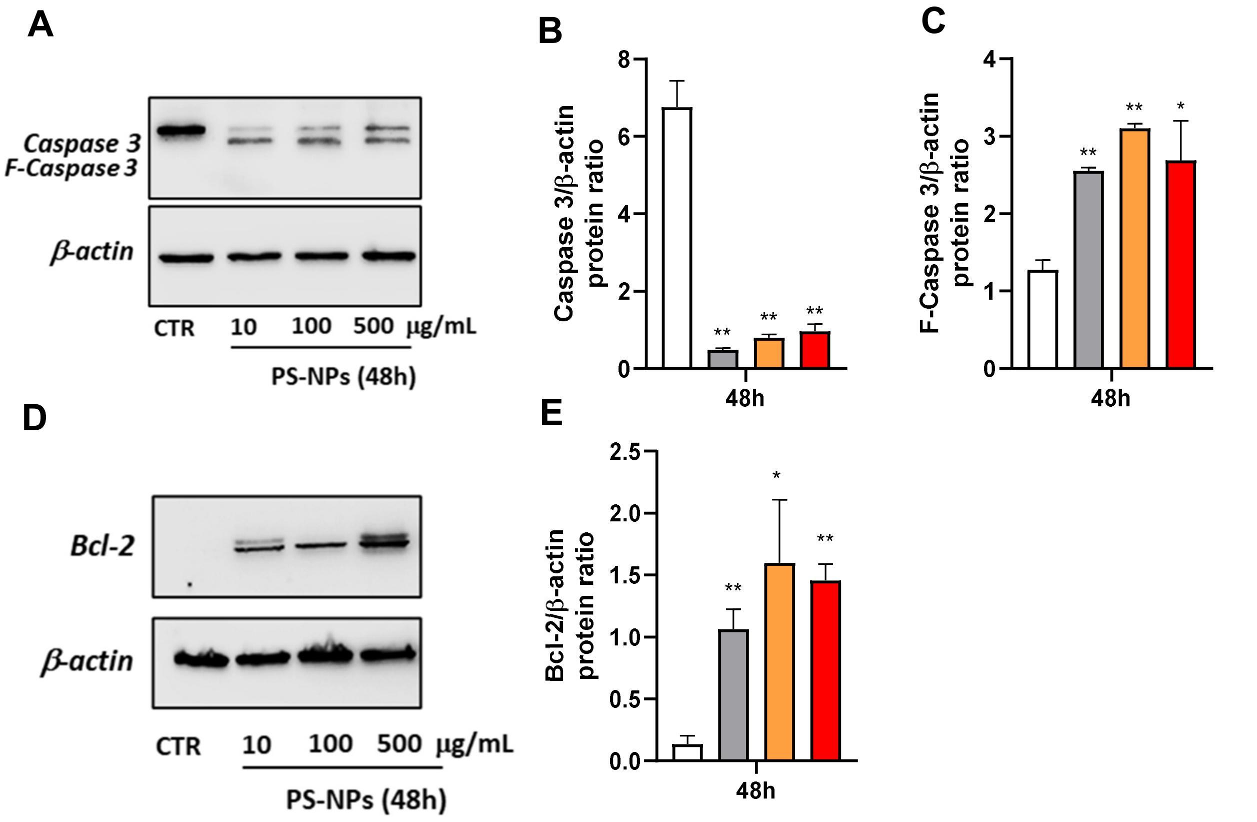

Supplementary Figure 5. (A) Western blot analysis of caspase 3 protein levels and its fragmented form (F-caspase 3) in A549 cells (500.000/well) untreated (CTR) or treated with PS-NPs (10, 100 and 500 µg/ml) for 48h. Caspase 3 and F-caspase 3 expression levels were normalized to β-actin protein expression. (B-C) Data are reported as ratio between optical density values of caspase 3 or F-caspase 3 immunoreactive bands to that of β-actin bands. (D) Immunoblot of Bcl-2 protein levels in A549 cells untreated (CTR) or treated with PS-NPs (10, 100 and 500 µg/ml) for 48h. Bcl-2 expression level was normalized to β-actin protein expression. (E) Data are reported as ratio between optical density values of Bcl-2 immunoreactive bands to that of β-actin bands. (B, C, E) All data are presented as mean ± SD of at least three independent experiments; P values are expressed as $^*P \le 0.05$ or $^{**} P \le 0.01$ vs. CTR.

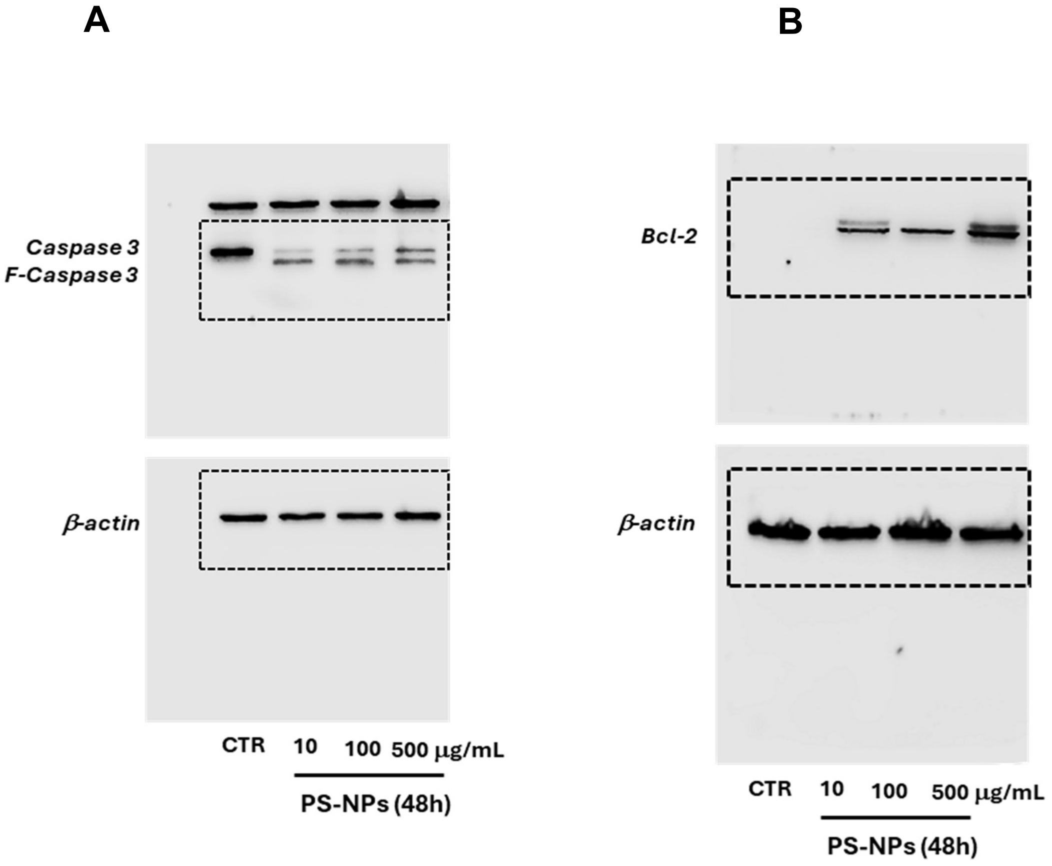

Supplementary Figure 6. Uncropped gels of Supplementary figure 5

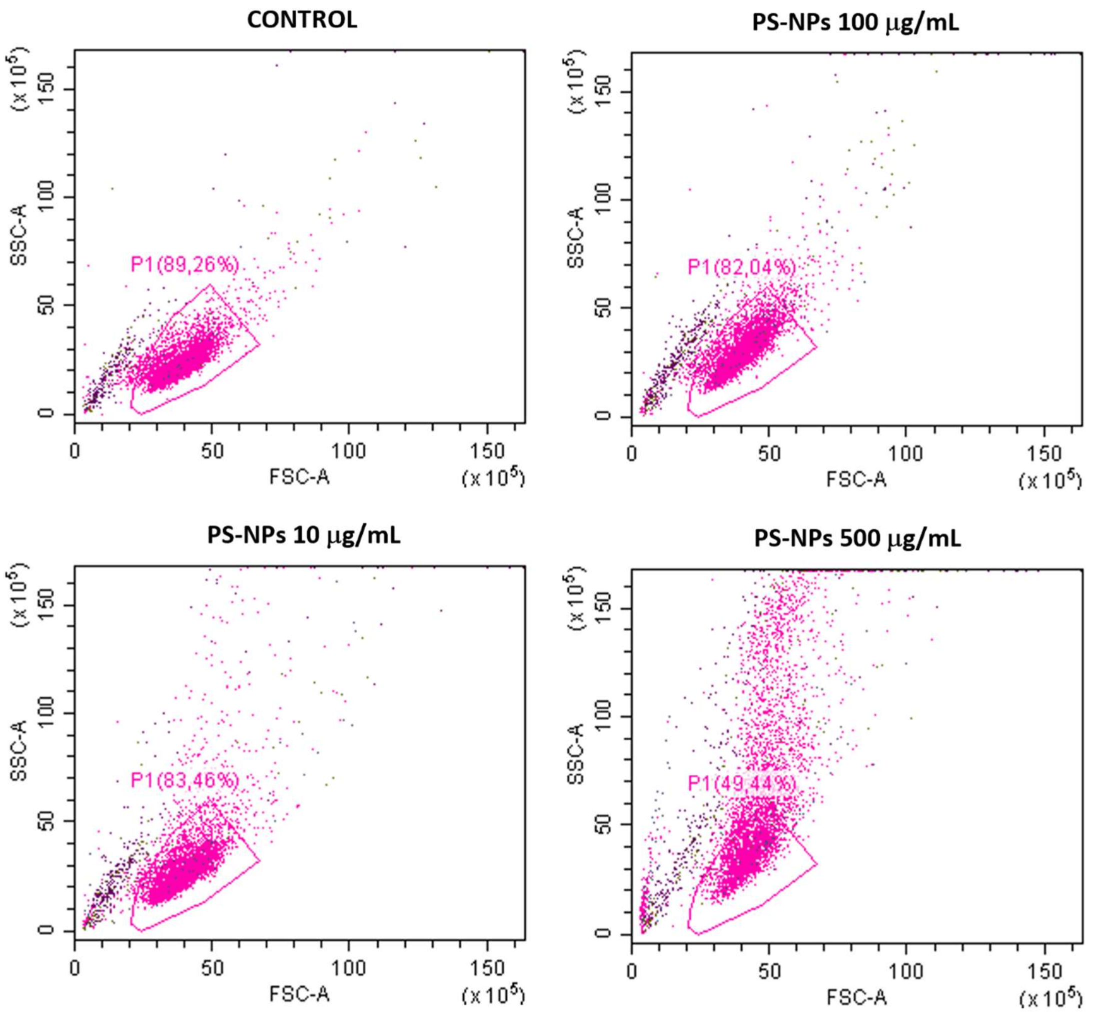

Supplementary Figure 7. Effect of PS-NPs on side scatter in A549 cells. Dot plots of the morphological parameters (FSC and SSC) of A549 cells not exposed (CONTROL) or exposed to selected concentrations of PS-NPs (10, 100, and 500μg/mL) for 48 h.
